# Phytase and nutrient-energy matrix: a strategic approach to enhancing the performance of broiler chickens fed a corn-soybean meal-based diet

**DOI:** 10.5713/ab.24.0565

**Published:** 2024-10-28

**Authors:** Pok Su Choi, Habeeb Tajudeen, Jun Young Mun, Sang Hun Ha, Abdolreza Hosseindoust, Serin Park, Hye In Park, Priscilla Neves Silvestre, Anushka Lokhande, Santosh Ingale, Jin Soo Kim

**Affiliations:** 1 Department of Animal Industry Convergence, Kangwon National University, Chuncheon 24341, Korea; 2 Advanced Enzymes Technologies Ltd, Thane, Maharashtra 400604, India

**Keywords:** Animal Nutrition, Animal Physiology, Animal Production, Broilers, Digestion

## Abstract

**Objective:**

This study examined the effects of a nutrient matrix with or without phytase on the performance of broiler chicken.

**Methods:**

A total of 2,000 day-old Ross 308 broiler chickens were assigned to 5 dietary treatments, with 10 broilers per replicate and 40 replicates per treatment. The experimental diets included 1. CON: A corn and soybean meal (SBM)-basal diet without phytase. 2, NC1: A corn-SBM-based diet with reduced nutrients, specifically 0.13% less phosphorus, 40 kcal/kg less metabolizable energy (ME), and 0.30% less crude protein (CP), without phytase. 3, NC1+PHYT: NC1+500 FTU/kg phytase. 4, NC2: Another corn-SBM-based diet with greater nutrient reductions, including 0.16% less phosphorus, 55 kcal/kg less ME, and 0.45% less CP, without phytase. 5, NC2+PHYT: NC2+1,000 FTU/kg phytase.

**Results:**

In the pre-starter and overall phase, feed conversion ratio (FCR) was higher in NC2 and NC2+PHYT. In the starter phase, body weight gain (BWG) was lower in NC2 and NC2+PHYT. In the grower phases, BWG was lower in NC2, while FCR was higher. At d28, the digestibility of ash was higher in NC1+PHYT, while the digestibility of Ca and phosphorus were higher in NC1+PHYT and NC2+PHYT. At day 42, the digestibility of ash, Ca, and phosphorus were higher in NC1+PHYT and NC2+PHYT. The level of tibia ash was lower in NC2. The level of myo-inositol was lower in NC2 at d28, while the level of myo-inositol at d42 was lower in NC1 and NC2.

**Conclusion:**

We concluded NC1+PHYT showed a higher growth performance comparable to CON, as against the lower performance observed in NC2, NC2+PHYT, and NC1.

## INTRODUCTION

Incorporating adequate crude protein (CP), energy, and phosphorus in broiler diets is essential but costly, prompting the use of dietary techniques to optimize cost and nutrient utilization. The addition of exogenous enzymes along with nutrient matrix, to corn-soybean meal (SBM) diets allows for nutritional substitution. This supplementation enhances performance and reduces endogenous losses at various stages of growth [1]. This is achieved by modifying the effect of phytase on the nutrient heterogeneity, enabling a counterreaction on antinutritional phytate and improving the accuracy in feed formulation [1]. Phytic acid, also known as inositol hexaphosphate IIP6 is a compound made of robust ionizable groups of phosphate found in plant components [2]. This is attributed to its molecule lacking preference as they form insoluble conjugates in the intestine, limiting the availability of cations, carbohydrates, protein, and enzymes such as trypsin and chymotrypsin, regarded as antinutritional agents [3]. Numerous studies have confirmed that the supplementation of phytase is proportional to the elimination of phosphorus, the release of minerals, proteins, and carbohydrates via its hydrolysis leading to an enhanced absorption of nutrients [4]. The nutritional structure of phytase also indicates that the quantity of calcium (Ca), phosphorus, metabolizable energy (ME), and CP availability in feed ingredients, the age of the birds, and the level of the enzyme influences its potency in the diets of broilers [2,5]. Hens administered lower quantities of inorganic Ca and phosphorus in their diets had greater ability to hydrolyze phytate rather than those provided higher amounts of these minerals [6]. In addition, the chelating effect of phytic acid found in corn-SBM stimulates a negative reaction with proteins and carbohydrates thereby impacting digestibility, metabolism, and cell structure [7]. Despite numerous studies focusing on the use of lower calcium levels, there has been limited attention on the combined effects of reduced phosphorus, CP, and ME levels when incorporating phytase in broiler diets. This gap is significant, as optimizing nutrient levels beyond calcium could further improve both cost-efficiency and nutrient absorption. Moreover, little is known about how these factors resonate with the enzymatic processes within phytase supplementation in modern broiler strains, particularly Ross 308.

Thus, the objective of this study will be to evaluate the effect of nutrient matrix with or without phytase on the growth performance, mortality rate, nutrient digestibility, tibia ash, and blood myo-inositol of Ross 308 broiler chicken fed corn-SBM based diet for 42 days.

## MATERIALS AND METHODS

### Animal care and ethical statement

The animal care and experimental techniques utilized in this study were approved by the Institutional Animal Care and Use Committee of Kangwon National University (Approval code b: KW-220413-1).

### Phytase information

The phytase enzyme known as DigePhos 10G evaluated in our experiment is a dry granulated strain of *Escherichia coli* (*E. coli*) bacteria produced by Advanced Enzyme Technologies Ltd. (Thane, India) with 6-phytase as the active element. The enzyme was synthesized via the controlled fermentation procedure adopting a unique Pichia pastoris strain. The 6-phytase is distinguished by a specific multi-layer film that includes an insulating layer for heat resistance, a phytase stabilizing reagent, and a unique hydrophobic layer with two significant properties of enduring high temperatures of up to 85°C stability for 45 to 60 seconds. The granules are homogeneous in size with colors ranging from cream to off-white. They play a vital role in facilitating the breakdown of indigestible phytic acid (phytate) existing in grains and oilseeds, especially at the L-6 locations. This mechanism improves the availability of important nutrients such as phosphorus, Ca, and other dietary components. Furthermore, each kilogram of the product contains 10 million units of protease activity (10,000,000 FTU/kg) such that, when included in diets, the 6-phytase shows an outstanding 90% recovery rate, resulting in a reduction in phosphorus excretion into the environment.

### Animals and experimental design

A total of 2,000 day-old Ross 308 broilers chicken were assigned to 5 dietary treatments, with 10 broilers per replicate and 40 replicates per treatment in a completely randomized design. Chickens were housed in a floor pen with rice husks as beddings and a temperature of 34°C for 1 to 3 days which was subsequently reduced to 23°C and constant lightening of 23 h/d in accordance with Ross 308 standard management requirement. The pen was equipped with a feeding trough and two nipple drinkers each with feed and water made available *ad-libitum*, and the rooms were heated by an electric brooding heater (JKBL210S / JKBL300S; JK Lighting Co., Ltd., Gyeonggi-do, Korea). The experiment was conducted for 42 days using a 3-phase feeding program with days 1 to 7 as the pre-starter phase, 8 to 14 as the starter phase/phase 1, days 15 to 28 as the grower phase/phase 2, and days 29 to 42 as the finisher phase/phase 3. The dietary treatments include 1. CON: A corn and SBM-basal diet without phytase. 2, NC1: A corn-SBM-based diet with reduced nutrients, specifically 0.13% less phosphorus, 40 kcal/kg less ME, and 0.30% less CP, without phytase. 3, NC1+PHYT: NC1+500 FTU/kg phytase. 4, NC2: Another corn-SBM-based diet with greater nutrient reductions, including 0.16% less phosphorus, 55 kcal/kg less ME, and 0.45% less CP, without phytase. 5, NC2+PHYT: NC2+1,000 FTU/kg phytase. The experimental diets were fed in a mash form and were subsequently substituted with pellet diets until 42 days. All diets met or exceeded the nutrient requirements recommended Ross 308 [8] management guide as shown in Table 1. Parameters such as feed intake (FI), average daily feed intake (ADFI) feed conversion ratio (FCR), body weight gain (BWG), average daily gain (ADG), mortality rate, nutrient digestibility (dry matter (DM), CP, gross energy (GE), ash, phosphorus, amino acid (AA), tibia ash, and blood myo-inositol were measured at the end of the experiments.

### Sample collection and chemical analysis

#### Growth performance

The growth indicators such as FI, BWG, ADG, ADFI, and FCR were measured for all birds at the initiation and the conclusion of each phase. The quantity of feed served was measured daily, and the amount of feed left in the feeders were noted at the end of each phase. The rate of mortality was also recorded from the inception of the experiment until the last phase.

### Nutrient digestibility

Nutrient balance trials were conducted at day 28 and 42 to determine the apparent ileal and total digestibility of DM, CP, AA, GE, calcium (Ca), and phosphorus. Two birds from each replicate were employed to facilitate the collection of excreta samples. The diets containing 0.25% chromium oxide as an indigestible marker were given, and excreta samples of 100 g/d per bird were collected from each bird. The feed and fecal samples were dried in a forced air-drying oven at 60°C for 72 h and ground in a Wiley laboratory mill (Thomas Model 4 Wiley Mill; Thomas Scientific, Swedesboro, NJ, USA) using a 1-mm screen.

The total nutrient digestibility was calculated as:


$$\begin{array}{l} \text{Total nutrient digestibility}\;(\%)=100-[100\times (\%\;\text{chromium in feed}/\\ \%\;\text{chromium in excreta})\times (\%\;\text{nutrient in excreta}/\%\;\text{nutrient in feed})] \end{array}$$

Experimental diets, and excreta samples were analyzed in triplicate for DM, method 930.15; AOAC [9], CP method 990.03; AOAC [9], and ash method 942.05; AOAC [9]. The Ca, and phosphorus (Method 985.01; AOAC [9]. The GE were measured by a bomb calorimeter (Model 1261; Parr Instrument Co., Moline, IL, USA), AA composition was evaluated using high-performance liquid chromatography (HPLC) [10], and chromium concentration was determined with an automated spectrophotometer (Jasco V-650; Jasco Corp., Tokyo, Japan) according to the procedure of Fenton and Fenton [11].

### Tibia ash and blood inositol

The left tibia bones were cleaned of all soft tissues, fibula bones, and cartilage caps for the purpose of tibia ash measurement. The tibias were then dried for 48 hours at 103°C and defatted. Following this, they were ashed overnight in a muffle furnace (Nabertherm L 40/11/B170; Nabertherm GmbH, Bremen, Germany) at 600°C in porcelain crucibles. After cooling in a desiccator, the percentage of tibia ash was determined.

To determine blood myo-inositol, approximately 2.5 mL samples of blood were drawn from 5 birds per treatment through the brachial wing vein on day 28 and day 42. It was then transferred to centrifugation at 1,500×g at 4°C. At the conclusion of the centrifugation process, the samples were gathered in a disposable vacutainer containing heparin sodium and centrifuged at 5,000×g for 15 min at 4°C. The supernatant from the samples was transferred to a 1 mL tube and stored at −20°C until analysis. The frozen samples were thawed and a volume of it was added to 2 volumes of perchloric acid using the methods described by Lee et al [12]. The sample was then incubated at 4°C for 30 minutes before centrifugation at 17,500×g for 10 minutes, and the supernatant was transferred to another clean tube for HPLC-pulsed amperometry.

### Statistical analysis

The data obtained in this study were statistically analyzed in a completely randomized design using the general linear model (GLM) procedure of SAS (SAS Institute Inc., Cary, NC, USA). Each time significant differences between treatment means were noticed, they were distinguished using Tukey’s Honestly Significant Difference test. Values of p<0.05 were considered significant.

## RESULTS

### Growth performance and mortality rate

The effects of phytase supplementation on growth performance and mortality rate of broiler chicken were shown in Tables 2, 3 respectively. During the pre-starter and the overall phase, the FCR was higher (p<0.05) in NC2 and NC2+PHYT compared with NC1+PHYT and CON. In the starter phase, BWG was lower (p<0.05) in NC2 and NC2+PHYT compared with CON. In the grower phase, BWG was lower (p<0.05) in NC2 compared with NC1+PHYT and CON, and FCR was higher (p<0.05) in NC2 compared with NC1+PHYT and CON. There was no significant difference in BWG, FI, and FCR in the finisher phase. There was no significant difference in the mortality rate across all treatments and phases.

### Nutrient and amino acid digestibility

The effect of phytase on the nutrient and AA digestibility of broiler chicken is shown in Tables 4, 5 respectively. At d 28, the digestibility of ash was higher (p<0.05) in NC1+PHYT compared with other treatments, while the digestibility of Ca and phosphorus were higher (p<0.05) in NC1+PHYT and NC2+PHYT compared with NC1 and NC2. At d 42, the digestibility of ash, Ca, and phosphorus were higher (p<0.05) in NC1+PHYT and NC2+PHYT compared with NC1 and NC2. There was no significant difference in the digestibility of all non-dispensable and dispensable AAs across all treatments.

### Tibia ash and blood inositol

The effect of phytase on the tibia ash and blood inositol is shown in Table 6. The level of tibia ash was lower (p<0.05) in NC2 compared with NC1+PHYT, NC1, and CON. At d 28, the level of myo-inositol was lower (p<0.05) in NC2 compared with other treatments, while the level of myo-inositol at d 42 was higher (p<0.05) in NC1+PHYT, NC2+PHYT, and CON compared with NC1.

## DISCUSSION

Assessing phytase matrix values can be challenging, especially when incorporating phytase at the standard level, or levels higher than normal inclusion standards [13,14]. However, the practicality of the strategy employed in this study is demonstrated by the fact that it showed similar BWG in NC1+PHYT comparable to the CON in the starter and grower phase, while FCR was higher in NC2, NC2+PHYT, and NC1 in the pre-starter phase. The FCR was also higher in NC2 in the grower phase, while in the overall phase, FCR was higher in NC2 and NC2+PHYT. Our result is a proof that a proper phytase supplementation in corn-SBM diets may improve the performance of broiler chicken by disintegrating the antinutritional factors present in the plant’s cell wall [15], thus, enhancing the better nutrient absorption observed in our study. This improvement occurs primarily due to the ability of phytase to release phosphorus from phytate, as this form of phosphorus is usually indigestible by chickens [16]. Phytase breaks down phytate, a complex molecule found in plant-based corn-SBM feed ingredients into simpler, and more readily absorbable forms of phosphorus by cleaving orthophosphate groups from the inositol ring of phytic acid, resulting in the release of free inorganic phosphorus and a series of intermediate myo-inositol phosphates [14]. Phytase increases phosphorus availability for bone development and helps reduce phosphorus pollution in the environment through the breakdown of bound phosphorus in plant-derived feed ingredients [13,17]. This dietary phosphorus is crucial for various physiological processes, including nutrient absorption, bone development, protein efficiency and energy metabolism.

Despite the improvement noticed in BWG in NC2+PHYT in the grower phase of this study, the performance of broiler chickens were higher in the NC1+PHYT. The influence of microbial phytase on inorganic phosphorus release is dependent on dietary phytate concentration, mineral concentrations, the specific dietary need of the animal being fed, and the phytase dose [17]. Although NC2+PHYT had higher phytase-supplemented at 1,000 FTU/kg, the reduction in phosphorus, CP and ME were less severe in NC1+PHYT as proven by the better performance in NC1 compared with NC2. This interprets that broilers in group NC1+PHYT were receiving a diet that is closer to the nutrient requirements of the Ross 308 broilers compared with NC2+PHYT. We, therefore, propose that NC1+PHYT was able to perform better than NC2+PHYT due to its 500 FTU/kg level of phytase that coincides with the nutrient matrix in the treatment to effectively enhance better bone ash and nutrient digestibility similar to the CON diet with a complete nutrient.

The higher tibia ash and increased blood inositol observed in the phytase-supplemented diets, especially at NC1+PHYT of this study was expected as the content of phosphorus released by phytase in corn-SBM diet is proportional to the bone ash content [18,19]. Birds fed reduced phosphorus, CP and ME below the Ross 308 standard nutrient recommendations showed a reduction in the quantity of tibia ash as seen in NC2. This is consistent with the study of Gautier et al [13] Dersjant-Li et al [14] where birds fed corn-SBM diets with low phosphorus and other nutrients without phytase had poor tibia ash quantity. However, the addition of phytase led to an improvement in the tibia ash. In the present study, the supplementation of phytase at NC1+PHYT numerically restored the initial tibia ash levels to a similar level with the CON diets, while the tibia ash in NC2 was improved when phytase was supplemented at NC2+PHYT. Thus, confirming the distribution of phosphorus and Ca in the phytase-supplemented diets.

Myo-inositol is a vital constituent of cellular phosphoinositide contributing to a range of cellular processes [20]. It is believed to aid in protein synthesis, implying that it may synchronize the transportation of protein, accounting for the enhancements in growth performance associated with higher blood Myo-inositol [20–23]. Besides the ability to manufacture myo-inositol from glucose, the body can also obtain it from diets through absorption in the digestive system or by the release of cellular phospholipids [21–24]. As a result, it is also feasible to speculate that a viable reason for the growth performance experienced in the phytase-supplemented diets arises from its ability to induce the concentration of Myo-inositol which reduces the liberation of insp6 allowing room for enough metabolic impacts in broiler chicken as shown in our study.

## CONCLUSION

Our experiment confirms that a moderate reduction in phosphorus, CP, and ME in broiler diets supplemented with an *E. coli* synthesized phytase (NC1+PHYT) results in a better performance compared to the NC2+PHYT diet, which had a greater reduction of these nutrients. Although, both treatments NC1+PHYT and NC2+PHYT improved phosphorus bioavailability, the effect was more pronounced in the NC1+PHYT diet containing basic feed −0.13% less phosphorus, −40 kcal/kg less ME, −0.30% less CP+500 FTU/kg phytase. The carryover effects of this diet were evident in the lower FCR, higher tibia ash content, and increased Myo-inositol levels, which contributed to the higher nutrient digestibility and BWG. These findings offer a practical approach for the poultry industry to optimize feed formulations by potentially reducing overall feed costs without compromising performance. This approach presents an economically viable solution for enhancing profitability in poultry production.

## Figures and Tables

**Table 1:** Ingredient and chemical composition of basal diet (as-fed basis)

Item	Phase 1			Phase 2			Phase 3		
		
	CON^1)^	NC1	NC2	CON	NC1	NC2	CON	NC1	NC2
Ingredient (%)	100.00	100.00	100.00	100.00	100.00	100.00	100.00	100.00	100.00
Corn	55.03	57.17	58.53	57.86	59.67	60.86	63.30	65.71	66.96
Soybean meal (45%)	36.06	35.21	34.67	36.37	35.55	35.02	31.21	29.95	29.11
Fish meal	3.00	3.00	3.00	-	-	-	-	-	-
Soy oil	3.00	2.01	1.35	3.00	2.13	1.56	3.00	1.95	1.57
Monocalcium phosphate	0.54	0.29	0.13	0.50	0.38	0.29	0.13	0.03	-
Limestone	1.37	1.37	1.37	1.27	1.27	1.27	1.36	1.36	1.36
Salt	0.30	0.30	0.30	0.30	0.30	0.30	0.30	0.30	0.30
L-Lysine·HCl (78%)	0.03	0.03	0.03	0.10	0.10	0.10	0.10	0.10	0.10
DL-Methionine (99%)	0.27	0.27	0.27	0.25	0.25	0.25	0.25	0.25	0.25
L-Threonine (99%)	0.10	0.10	0.10	0.10	0.10	0.10	0.10	0.10	0.10
Vitamin and mineral premix^2)^	0.25	0.25	0.25	0.25	0.25	0.25	0.25	0.25	0.25
Calculated composition (%)									
Metabolizable energy (kcal/kg)	3,000	2,960	2,945	3,100	3,060	3,045	3,200	3,160	3,145
Crude protein	23.00	22.70	22.55	21.50	21.20	21.05	19.50	19.20	19.05
Total calcium	0.95	0.95	0.95	0.80	0.80	0.80	0.70	0.70	0.70
Digestible phosphorus	0.54	0.41	0.39	0.44	0.32	0.29	0.36	0.24	0.21
Digestible lysine	1.35	1.35	1.35	1.18	1.18	1.18	1.11	1.11	1.11
Digestible methionine+cysteine	1.00	1.00	1.00	0.92	0.92	0.92	0.86	0.86	0.86

1) CON, basic feed; NC1, basic feed −0.13% phosphorus, −40 kcal/kg ME, −0.30% CP; NC1+PHYT, NC1+500 FTU/kg phytase; NC2, basic feed −0.160% phosphorus, −55 kcal/kg ME, −0.45% CP; NC2+PHYT, NC2+1,000 FTU/kg phytase.

2) Provided per kg of diet: 9,000 IU vitamin A (palmitate), 1,800 IU vitamin D_3_ (cholecalciferol), 30 mg vitamin E (dl-α-tocopheryl acetate), 1 mg vitamin K_3_ (menadione), 1 mg vitamin B_1_ (thiamin), 10 mg vitamin B_2_ (riboflavin), 4 mg vitamin B_6_ (pyridoxine), 0.02 mg vitamin B_12_ (cyanocobalamin), 30 mg niacin, 12 mg pantothenic acid, 0.50 mg folic acid, 0.20 mg biotin, 80 mg Fe, 20 mg Cu, 120 mg Mn, 1.40 mg I, and 0.30 mg Se.

**Table 2:** The effects of phytase supplementation level on growth performance in broiler chicken

Item	CON^1)^	NC1	NC1+PHYT	NC2	NC2+PHYT	SEM	p-value
Pre-starter (d 1–7)							
BWG (g)	126.30	122.03	125.12	115.96	118.13	1.408	0.088
FI (g)	93.08	93.69	93.55	88.99	91.04	1.320	0.773
FCR	1.10^c^	1.16^b^	1.11^c^	1.19^a^	1.19^a^	0.013	0.047
Starter (d 8–14)							
BWG (g)	399.83^a^	372.44^ab^	381.56^ab^	361.36^b^	369.86^b^	3.967	0.021
FI (g)	330.37	307.33	310.05	302.83	308.76	5.607	0.589
FCR	1.22	1.23	1.22	1.24	1.23	0.019	0.096
Grower (d 15–28)							
BWG (g)	1,338.01^a^	1,297.55^ab^	1,316.60^a^	1,275.94^b^	1,297.89^ab^	6.570	0.032
FI (g)	1,460.90	1,452.93	1,459.32	1,443.79	1,454.83	10.735	0.990
FCR	1.55^b^	1.57^ab^	1.56^b^	1.58^a^	1.57^ab^	0.010	0.036
Finisher (d 29–42)							
BWG (g)	2,326.61	2,277.40	2,301.67	2,244.56	2,270.28	9.995	0.091
FI (g)	1,706.98	1,703.11	1,705.36	1,706.47	1,702.26	14.232	0.899
FCR	1.73	1.74	1.73	1.77	1.75	0.013	0.069
Overall (d 1–42)							
ADG (g)	54.42	53.25	53.82	52.46	53.07	0.238	0.092
ADFI (g)	85.50	84.69	84.96	84.34	84.69	0.387	0.917
FCR	1.57^b^	1.59^ab^	1.58^b^	1.61^a^	1.60^a^	0.007	0.027

SEM, standard error of means; BWG, body weight gain; FI, feed intake; FCR, feed conversion ratio; ADG, average daily gain; ADFI, average daily feed intake.

1) CON, basic feed; NC1, basic feed −0.13% phosphorus, −40 kcal/kg ME, −0.30% CP; NC1+PHYT, NC1+500 FTU/kg phytase; NC2, basic feed −0.160% phosphorus, −55 kcal/kg ME, −0.45% CP; NC2+PHYT, NC2+1,000 FTU/kg phytase.

a–c Means with different superscript within a row are significantly different (p<0.05).

**Table 3:** The effects of phytase supplementation level on mortality in broiler chicken

Item	CON^1)^	NC1	NC1+PHYT	NC2	NC2+PHYT	SEM	p-value
Mortality (%)							
Pre starter (d 1–7)	0.400	0.400	0.300	0.400	0.350	0.112	0.896
Starter (d 8–14)	0.509	0.612	0.560	0.613	0.508	0.156	0.556
Grower (d 15–28)	0.517	0.628	0.521	0.622	0.521	0.160	0.544
Finisher (d 29–42)	0.619	0.639	0.529	0.643	0.538	0.163	0.508
Overall (d 1–42)	2.045	2.279	2.010	2.279	1.917	0.288	0.608

SEM, standard error of means.

1) CON, basic feed; NC1, basic feed −0.13% phosphorus, −40 kcal/kg ME, −0.30% CP; NC1+PHYT, NC1+500 FTU/kg phytase; NC2, basic feed −0.160% phosphorus, −55 kcal/kg ME, −0.45% CP; NC2+PHYT, NC2+1,000 FTU/kg phytase.

a–c Means with different superscript within a row are significantly different (p<0.05).

**Table 4:** The effects of phytase supplementation level on nutrient digestibility in broiler chicken (%)

Item	CON^1)^	NC1	NC1+PHYT	NC2	NC2+PHYT	SEM	p-value
Day 28							
DM	72.81	72.14	72.60	71.93	72.10	0.456	0.091
CP	66.76	65.90	66.60	65.77	66.39	0.437	0.089
GE	76.43	76.22	76.33	76.23	76.38	0.680	0.790
EE	85.02	84.92	85.01	84.94	85.02	0.465	0.849
Ash	43.85^b^	40.98^c^	45.39^a^	40.48^c^	44.48^b^	0.406	<0.001
Ca	70.97^bc^	69.99^c^	73.25^a^	69.63^c^	72.47^ab^	0.789	<0.001
P	46.49^b^	45.82^b^	49.04^a^	45.40^b^	48.84^a^	0.497	<0.001
Day 42							
DM	73.53	73.13	73.35	72.49	72.98	0.396	0.052
CP	68.95	68.44	68.80	68.35	68.64	0.488	0.278
GE	76.21	76.05	76.25	76.02	76.28	0.665	0.737
EE	84.63	84.53	84.52	84.40	84.58	0.482	0.675
Ash	44.93^a^	42.11^b^	45.23^a^	41.66^b^	44.67^a^	0.527	<0.001
Ca	72.09^bc^	71.11^cd^	74.32^a^	70.76^d^	73.13^ab^	0.455	<0.001
P	47.97^bc^	47.18^c^	50.24^a^	46.90^c^	49.45^ab^	0.529	<0.001

SEM, standard error of means; DM, dry matter; CP, crude protein; GE, gross energy; EE, ether extract; Ca, calcium; P, phosphorus.

1) CON, basic feed; NC1, basic feed −0.13% phosphorus, −40 kcal/kg ME, −0.30% CP; NC1+PHYT, NC1+500 FTU/kg phytase; NC2, basic feed −0.160% phosphorus, −55 kcal/kg ME, −0.45% CP; NC2+PHYT, NC2+1,000 FTU/kg phytase.

a–d Means with different superscript within a row are significantly different (p<0.05).

**Table 5:** The effects of phytase supplementation level on amino acid digestibility in broiler chicken (%)

Item	CON^1)^	NC1	NC1+PHYT	NC2	NC2+PHYT	SEM	p-value
Non-dispensable							
Arginine	86.76	83.92	87.97	83.58	87.34	0.654	0.096
Histidine	78.46	78.47	80.22	78.60	80.72	0.677	0.083
Isoleucine	80.87	77.81	80.07	77.77	79.93	0.774	0.181
Leucine	81.29	78.16	80.07	77.68	79.44	0.445	0.266
Lysine	80.05	78.42	79.62	77.46	79.09	0.728	0.093
Methionine	83.79	80.21	82.40	79.59	81.82	0.495	0.123
Phenylalanine	72.76	72.46	73.60	71.74	72.29	0.738	0.176
Threonine	77.81	74.37	76.06	73.70	75.42	0.569	0.161
Tryptophan	79.51	78.69	80.58	78.61	79.87	0.446	0.202
Valine	78.83	76.763	78.16	76.34	77.86	0.516	0.090
Dispensable							
Alanine	79.69	76.49	79.05	76.49	78.58	0.517	0.176
Aspartic acid	78.56	76.73	77.45	76.97	77.96	0.481	0.155
Cysteine	66.62	61.89	65.20	61.41	64.24	0.752	0.102
Glutamic acid	80.01	76.66	78.71	76.79	77.38	0.532	0.097
Glycine	71.03	69.02	70.66	68.77	69.51	0.602	0.336
Proline	68.08	64.71	66.47	64.40	65.51	0.702	0.236
Serine	75.98	74.53	75.90	74.46	75.43	0.584	0.633
Tyrosine	80.36	77.75	78.75	77.23	78.84	0.847	0.332

SEM, standard error of means.

1) CON, basic feed; NC1, basic feed −0.13% phosphorus, −40 kcal/kg ME, −0.30% CP; NC1+PHYT, NC1+500 FTU/kg phytase; NC2, basic feed −0.160% phosphorus, −55 kcal/kg ME, −0.45% CP; NC2+PHYT, NC2+1,000 FTU/kg phytase.

a–c Means with different superscript within a row are significantly different (p<0.05).

**Table 6:** The effects of phytase supplementation level on tibia ash and blood myo-inositol in broiler chicken

Item	CON^1)^	NC1	NC1+PHYT	NC2	NC2+PHYT	SEM	p-value
Tibia ash (%)	51.03^a^	50.20^a^	50.45^a^	48.74^b^	49.13^ab^	0.055	0.021
Myo-inositol (μM)							
d 28	253.82^a^	245.03^ab^	247.56^ab^	233.25^c^	242.36^b^	3.453	0.004
d 42	259.22^a^	243.57^b^	254.23^a^	241.11^b^	248.60^ab^	3.372	0.005

SEM, standard error of means.

1) CON, basic feed; NC1, basic feed −0.13% phosphorus, −40 kcal/kg ME, −0.30% CP; NC1+PHYT, NC1+500 FTU/kg phytase; NC2, basic feed −0.160% phosphorus, −55 kcal/kg ME, −0.45% CP; NC2+PHYT, NC2+1,000 FTU/kg phytase.

a–c Means with different superscript within a row are significantly different (p<0.05).
